# Bis[3-dimethyl­amino-1-(pyridin-2-yl)prop-2-en-1-one-κ^2^ *N* ^1^,*O*]tris­(nitrato-κ^2^ *O*,*O*)gadolinium(III) ethanol disolvate

**DOI:** 10.1107/S1600536811050458

**Published:** 2011-11-30

**Authors:** Xia Liu, Dahua Hu

**Affiliations:** a Department of City Science, Jiangsu City Vocation College, Nanjing, 210003, People’s Republic of China

## Abstract

In the title compound, [Gd(NO_3_)_3_(C_10_H_12_N_2_O)_2_]·2C_2_H_5_OH, the Gd^III^ ion and one nitrate anion are located on a twofold rotation axis. The Gd^III^ ion is ten-coordinated by two N and two O atoms from two bidentate 3-(*N*,*N*-dimethyl­amino)-1-(2-pyrid­yl)prop-2-en-1-one) ligands and six O atoms from three nitrate anions in a distorted bicapped square-anti­prismatic geometry. In the crystal, the components are linked by O—H⋯O hydrogen bonds. The ethanol solvent mol­ecule is disordered over two positions in a ratio 0.615 (16):0.385 (16).

## Related literature

For isotypic structures, see: Hu (2010 ▶); Shen *et al.* (2011 ▶). For compounds containing the 3-(*N*,*N*-dimethyl­amino)-1-(2-pyrid­yl)prop-2-en-1-one) ligand, see: Bi (2009 ▶); Hu & Tian (2007 ▶); Li *et al.* (2005 ▶); Shen *et al.* (2011 ▶); Wang *et al.* (2005 ▶).
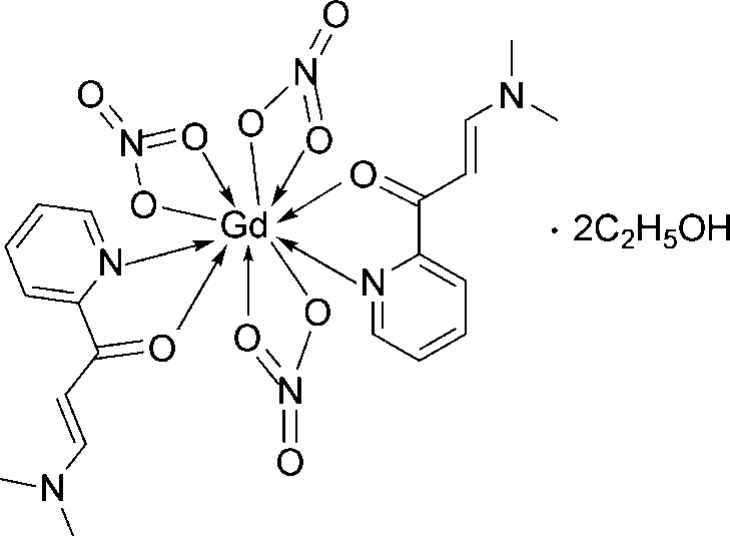


## Experimental

### 

#### Crystal data

[Gd(NO_3_)_3_(C_10_H_12_N_2_O)_2_]·2C_2_H_6_O
*M* *_r_* = 787.85
Monoclinic, 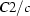

*a* = 21.322 (2) Å
*b* = 10.9876 (11) Å
*c* = 16.3844 (16) Å
β = 121.020 (2)°
*V* = 3289.5 (6) Å^3^
*Z* = 4
Mo *K*α radiation
μ = 2.09 mm^−1^
*T* = 293 K
0.25 × 0.22 × 0.18 mm

#### Data collection

Bruker SMART CCD area-detector diffractometer
Absorption correction: multi-scan (*SADABS*; Bruker, 2000 ▶) *T*_min_ = 0.624, *T*_max_ = 0.705
8248 measured reflections
3005 independent reflections
2563 reflections with *I* > 2σ(*I*)
*R*_int_ = 0.053

#### Refinement

*R*[*F* ^2^ > 2σ(*F* ^2^)] = 0.049
*wR*(*F* ^2^) = 0.087
*S* = 1.02
3005 reflections
237 parameters
130 restraints
H-atom parameters constrained
Δρ_max_ = 1.28 e Å^−3^
Δρ_min_ = −0.64 e Å^−3^

### 

Data collection: *SMART* (Bruker, 2000 ▶); cell refinement: *SAINT* (Bruker, 2000 ▶); data reduction: *SAINT*; program(s) used to solve structure: *SHELXS97* (Sheldrick, 2008 ▶); program(s) used to refine structure: *SHELXL97* (Sheldrick, 2008 ▶); molecular graphics: *SHELXTL* (Sheldrick, 2008 ▶); software used to prepare material for publication: *SHELXTL*.

## Supplementary Material

Crystal structure: contains datablock(s) I, global. DOI: 10.1107/S1600536811050458/br2183sup1.cif

Structure factors: contains datablock(s) I. DOI: 10.1107/S1600536811050458/br2183Isup2.hkl

Additional supplementary materials:  crystallographic information; 3D view; checkCIF report

## Figures and Tables

**Table 1:** Hydrogen-bond geometry (Å, °)

*D*—H⋯*A*	*D*—H	H⋯*A*	*D*⋯*A*	*D*—H⋯*A*
O7—H7*A*⋯O3	0.82	2.26	3.084 (12)	179
O7*B*—H7*B*⋯O3	0.82	2.19	3.00 (2)	168

**Fig. 1.**
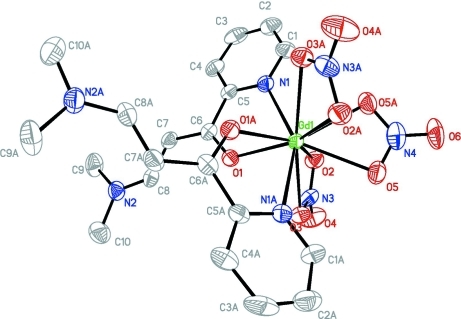
Molecular structure of the title compound, showing 30% probability displacement ellipsoids and the atom-numbering. The solvent molecule is omitted for clarity. symmetry operation i: -x, y, 0.5 - z.

## supplementary crystallographic information

### Comment

The crystal structures of some coordination complexes of the ligand 3-(*N*,*N*-dimethylamino)-1-(2-pyridyl)prop-2-en-1-one) with Co, Ni, Zn, Cd, and Pr, have been described ((Bi *et al.*, 2009; Hu & Tian, 2007; Li *et al.*, 2005; Wang *et al.*, 2005 Hu, 2010). Here we report the crystal structure of the title complex with gadolinium(III).

The coordination geometry about Gd(III) center is shown in Fig. 1. Each Gd(III) atom is in a ten coordinate environment comprising two oxygen atoms and two nitrogen atoms from the two bidentate organic ligands *L* and six oxygen atoms from three tertiary nitrate anions that act as bidentate anionic ligands. Thus the coordination polyhedron of Gd(III) is a distorted bicapped square antiprism. The Gd—O distances lie in two groups; those to the oxygen atoms of organic ligands are 2.348 Å, whereas those to nitrate O atoms are in the range of 2.494 (5)–2.549 (5) Å, which were similar to those in the Pr complex (Hu, 2010; Shen *et al.*, 2011).

### Experimental

All solvents and chemicals were of analytical grade and were used without further purification. For the synthesis of title compoud, a solution of ligand (0.2 mmol) and Gd(NO_3_)_3_ (0.1 mmol) in 50 ml e thanol was refluxed for 1 h, and then cooled to room temperature and filtered. Single crystals suitable for X-ray analysis were grown from the ethanol solution by slow evaporation at room temperature in air.

### Refinement

The ethanol solvent molecule is disordered over two orientations in a ratio 0.615 (16):0.385 (16). Restraints were applied to the displacement parameters and distances of the non-hydrogen atoms of the ethanol solvent molecule, respectively, in order to keep them structurely reasonable. The highest difference electron density peak of 1.28 e/Å^3^  and deepest hole of -0.64 e/Å^3^ were found to be 0.03 and 0.66 Å from the Gd1 atom, respectively. All the hydrogen atoms were geometrically positioned (C—H 0.93–0.97 Å and O—H 0.82 Å) and refined as riding, with *U*_iso_(H)=1.2–1.5 *U*_eq_ of the parent atoms.

### Crystal data

**Table:**

[Gd(NO_3_)_3_(C_10_H_12_N_2_O)_2_]·2C_2_H_6_O	*F*(000) = 1588
*M**_r_* = 787.85	*D*_x_ = 1.591 Mg m^−^^3^
Monoclinic, *C*2/*c*	Mo *K*α radiation, λ = 0.71073 Å
Hall symbol: -C 2yc	Cell parameters from 1903 reflections
*a* = 21.322 (2) Å	θ = 2.2–25.3°
*b* = 10.9876 (11) Å	µ = 2.09 mm^−^^1^
*c* = 16.3844 (16) Å	*T* = 293 K
β = 121.020 (2)°	Block, colorless
*V* = 3289.5 (6) Å^3^	0.25 × 0.22 × 0.18 mm
*Z* = 4	

### Data collection

**Table:**

Bruker SMART CCD area-detector diffractometer	3005 independent reflections
Radiation source: fine-focus sealed tube	2563 reflections with *I* > 2σ(*I*)
graphite	*R*_int_ = 0.053
phi and ω scans	θ_max_ = 25.3°, θ_min_ = 2.2°
Absorption correction: multi-scan (*SADABS*; Bruker, 2000)	*h* = −25→23
*T*_min_ = 0.624, *T*_max_ = 0.705	*k* = −11→13
8248 measured reflections	*l* = −13→19

### Refinement

**Table:**

Refinement on *F*^2^	Primary atom site location: structure-invariant direct methods
Least-squares matrix: full	Secondary atom site location: difference Fourier map
*R*[*F*^2^ > 2σ(*F*^2^)] = 0.049	Hydrogen site location: inferred from neighbouring sites
*wR*(*F*^2^) = 0.087	H-atom parameters constrained
*S* = 1.02	*w* = 1/[σ^2^(*F*_o_^2^) + (0.030*P*)^2^] where *P* = (*F*_o_^2^ + 2*F*_c_^2^)/3
3005 reflections	(Δ/σ)_max_ = 0.001
237 parameters	Δρ_max_ = 1.28 e Å^−^^3^
130 restraints	Δρ_min_ = −0.64 e Å^−^^3^

### Special details

**Table:**

Geometry. All e.s.d.'s (except the e.s.d. in the dihedral angle between two l.s. planes) are estimated using the full covariance matrix. The cell e.s.d.'s are taken into account individually in the estimation of e.s.d.'s in distances, angles and torsion angles; correlations between e.s.d.'s in cell parameters are only used when they are defined by crystal symmetry. An approximate (isotropic) treatment of cell e.s.d.'s is used for estimating e.s.d.'s involving l.s. planes.
Refinement. Refinement of *F*^2^ against ALL reflections. The weighted *R*-factor *wR* and goodness of fit *S* are based on *F*^2^, conventional *R*-factors *R* are based on *F*, with *F* set to zero for negative *F*^2^. The threshold expression of *F*^2^ > σ(*F*^2^) is used only for calculating *R*-factors(gt) *etc*. and is not relevant to the choice of reflections for refinement. *R*-factors based on *F*^2^ are statistically about twice as large as those based on *F*, and *R*- factors based on ALL data will be even larger.

### Fractional atomic coordinates and isotropic or equivalent isotropic displacement parameters (Å^2^)

**Table:**

	*x*	*y*	*z*	*U*_iso_*/*U*_eq_	Occ. (<1)
Gd1	0.5000	0.68895 (3)	0.7500	0.04053 (15)	
C1	0.3556 (3)	0.6948 (6)	0.5155 (4)	0.0635 (17)	
H1	0.3456	0.7663	0.5369	0.076*	
C2	0.3110 (4)	0.6640 (7)	0.4213 (4)	0.072 (2)	
H2	0.2723	0.7140	0.3799	0.086*	
C3	0.3250 (4)	0.5580 (8)	0.3900 (4)	0.077 (2)	
H3	0.2963	0.5349	0.3266	0.093*	
C4	0.3821 (3)	0.4859 (6)	0.4536 (4)	0.0626 (18)	
H4	0.3916	0.4123	0.4339	0.075*	
C5	0.4255 (3)	0.5237 (5)	0.5470 (3)	0.0455 (14)	
C6	0.4913 (3)	0.4574 (5)	0.6206 (4)	0.0478 (15)	
C7	0.5062 (3)	0.3384 (5)	0.6053 (4)	0.0547 (16)	
H7	0.4742	0.2988	0.5486	0.066*	
C8	0.5677 (4)	0.2802 (5)	0.6734 (4)	0.0551 (16)	
H8	0.5963	0.3232	0.7296	0.066*	
C9	0.5512 (4)	0.0952 (6)	0.5869 (5)	0.088 (2)	
H9A	0.5037	0.0784	0.5774	0.132*	
H9B	0.5772	0.0202	0.5962	0.132*	
H9C	0.5459	0.1363	0.5319	0.132*	
C10	0.6585 (4)	0.1229 (6)	0.7484 (5)	0.088 (2)	
H10A	0.6847	0.1863	0.7937	0.131*	
H10B	0.6883	0.0909	0.7252	0.131*	
H10C	0.6470	0.0590	0.7785	0.131*	
N1	0.4118 (2)	0.6285 (4)	0.5772 (3)	0.0490 (12)	
N2	0.5917 (3)	0.1716 (4)	0.6697 (4)	0.0614 (14)	
N3	0.6199 (3)	0.7461 (5)	0.7143 (4)	0.0601 (14)	
N4	0.5000	0.9580 (8)	0.7500	0.083 (3)	
O1	0.5330 (2)	0.5152 (3)	0.6968 (2)	0.0544 (11)	
O2	0.5542 (2)	0.7683 (4)	0.6534 (3)	0.0611 (12)	
O3	0.6327 (2)	0.7032 (3)	0.7932 (3)	0.0579 (11)	
O4	0.6696 (3)	0.7642 (5)	0.6994 (3)	0.0965 (17)	
O5	0.5524 (2)	0.8938 (4)	0.8143 (3)	0.0671 (12)	
O6	0.5000	1.0655 (6)	0.7500	0.098 (2)	
O7	0.7048 (8)	0.4494 (12)	0.8453 (9)	0.156 (6)	0.615 (16)
H7A	0.6849	0.5163	0.8315	0.234*	0.615 (16)
C11	0.7467 (10)	0.443 (2)	0.9319 (11)	0.136 (6)	0.615 (16)
H11A	0.7208	0.3934	0.9538	0.163*	0.615 (16)
H11B	0.7464	0.5250	0.9543	0.163*	0.615 (16)
C12	0.8233 (9)	0.4041 (19)	0.9907 (14)	0.130 (6)	0.615 (16)
H12A	0.8274	0.3454	1.0366	0.195*	0.615 (16)
H12B	0.8535	0.4733	1.0232	0.195*	0.615 (16)
H12C	0.8390	0.3682	0.9508	0.195*	0.615 (16)
O7B	0.7568 (13)	0.525 (2)	0.8763 (16)	0.153 (7)	0.385 (16)
H7B	0.7227	0.5721	0.8609	0.230*	0.385 (16)
C11B	0.773 (2)	0.475 (3)	0.9530 (18)	0.141 (8)	0.385 (16)
H11C	0.7290	0.4706	0.9554	0.169*	0.385 (16)
H11D	0.8060	0.5308	1.0032	0.169*	0.385 (16)
C12B	0.8080 (19)	0.353 (2)	0.979 (3)	0.133 (9)	0.385 (16)
H12D	0.7729	0.2944	0.9740	0.200*	0.385 (16)
H12E	0.8487	0.3549	1.0435	0.200*	0.385 (16)
H12F	0.8249	0.3314	0.9370	0.200*	0.385 (16)

### Atomic displacement parameters (Å^2^)

**Table:**

	*U*^11^	*U*^22^	*U*^33^	*U*^12^	*U*^13^	*U*^23^
Gd1	0.0412 (3)	0.0372 (2)	0.0343 (2)	0.000	0.01310 (17)	0.000
C1	0.051 (4)	0.072 (4)	0.052 (4)	0.006 (4)	0.016 (3)	0.004 (3)
C2	0.059 (5)	0.090 (6)	0.048 (4)	−0.007 (4)	0.015 (3)	0.008 (4)
C3	0.057 (5)	0.120 (7)	0.038 (4)	−0.027 (5)	0.012 (3)	−0.005 (4)
C4	0.059 (4)	0.074 (5)	0.052 (4)	−0.019 (4)	0.027 (3)	−0.020 (3)
C5	0.042 (4)	0.052 (4)	0.038 (3)	−0.012 (3)	0.017 (3)	−0.003 (3)
C6	0.050 (4)	0.051 (4)	0.046 (3)	−0.008 (3)	0.028 (3)	−0.005 (3)
C7	0.054 (4)	0.049 (4)	0.059 (4)	−0.006 (3)	0.027 (3)	−0.015 (3)
C8	0.064 (4)	0.047 (4)	0.062 (4)	−0.010 (3)	0.037 (3)	−0.012 (3)
C9	0.120 (7)	0.053 (4)	0.095 (5)	−0.001 (4)	0.057 (5)	−0.018 (4)
C10	0.085 (6)	0.073 (5)	0.100 (6)	0.021 (4)	0.044 (5)	0.004 (4)
N1	0.046 (3)	0.046 (3)	0.043 (3)	−0.002 (2)	0.015 (2)	−0.001 (2)
N2	0.068 (4)	0.049 (3)	0.072 (3)	0.002 (3)	0.039 (3)	−0.005 (3)
N3	0.055 (4)	0.067 (3)	0.059 (4)	−0.016 (3)	0.029 (3)	−0.018 (3)
N4	0.112 (9)	0.049 (6)	0.101 (7)	0.000	0.065 (7)	0.000
O1	0.053 (3)	0.047 (2)	0.047 (2)	0.0048 (19)	0.014 (2)	−0.0065 (19)
O2	0.058 (3)	0.067 (3)	0.048 (2)	−0.004 (2)	0.020 (2)	0.003 (2)
O3	0.055 (3)	0.065 (3)	0.045 (2)	−0.002 (2)	0.020 (2)	−0.002 (2)
O4	0.068 (4)	0.153 (5)	0.082 (3)	−0.023 (3)	0.049 (3)	−0.011 (3)
O5	0.074 (3)	0.049 (3)	0.074 (3)	−0.007 (2)	0.036 (3)	−0.009 (2)
O6	0.131 (7)	0.042 (4)	0.150 (7)	0.000	0.093 (6)	0.000
O7	0.147 (8)	0.135 (8)	0.138 (7)	0.024 (6)	0.039 (6)	−0.033 (6)
C11	0.137 (9)	0.136 (9)	0.137 (8)	0.007 (7)	0.072 (7)	−0.005 (7)
C12	0.132 (9)	0.117 (9)	0.127 (8)	0.005 (8)	0.057 (7)	0.001 (8)
O7B	0.146 (10)	0.157 (10)	0.164 (10)	0.004 (8)	0.086 (7)	0.008 (8)
C11B	0.135 (10)	0.140 (10)	0.144 (10)	0.002 (8)	0.071 (7)	−0.011 (8)
C12B	0.129 (11)	0.137 (11)	0.130 (10)	−0.011 (8)	0.064 (8)	0.006 (8)

### Geometric parameters (Å, °)

**Table:**

Gd1—O1	2.352 (4)	C9—H9A	0.9600
Gd1—O1^i^	2.352 (4)	C9—H9B	0.9600
Gd1—O5	2.492 (4)	C9—H9C	0.9600
Gd1—O5^i^	2.492 (4)	C10—N2	1.444 (7)
Gd1—O3	2.543 (4)	C10—H10A	0.9600
Gd1—O3^i^	2.543 (4)	C10—H10B	0.9600
Gd1—O2	2.547 (4)	C10—H10C	0.9600
Gd1—O2^i^	2.547 (4)	N3—O4	1.220 (6)
Gd1—N1	2.551 (4)	N3—O2	1.254 (6)
Gd1—N1^i^	2.551 (4)	N3—O3	1.267 (6)
Gd1—N3^i^	2.968 (6)	N4—O6	1.181 (9)
C1—N1	1.319 (7)	N4—O5^i^	1.281 (5)
C1—C2	1.374 (8)	N4—O5	1.281 (5)
C1—H1	0.9300	O7—C11	1.228 (9)
C2—C3	1.366 (9)	O7—H7A	0.8200
C2—H2	0.9300	C11—C12	1.469 (9)
C3—C4	1.374 (8)	C11—H11A	0.9700
C3—H3	0.9300	C11—H11B	0.9700
C4—C5	1.382 (7)	C12—H12A	0.9600
C4—H4	0.9300	C12—H12B	0.9600
C5—N1	1.344 (7)	C12—H12C	0.9600
C5—C6	1.487 (7)	O7B—C11B	1.242 (10)
C6—O1	1.268 (6)	O7B—H7B	0.8200
C6—C7	1.399 (7)	C11B—C12B	1.485 (10)
C7—C8	1.365 (8)	C11B—H11C	0.9700
C7—H7	0.9300	C11B—H11D	0.9700
C8—N2	1.311 (7)	C12B—H12D	0.9600
C8—H8	0.9300	C12B—H12E	0.9600
C9—N2	1.444 (7)	C12B—H12F	0.9600
			
O1—Gd1—O1^i^	71.45 (19)	C3—C4—C5	119.4 (6)
O1—Gd1—O5	137.36 (14)	C3—C4—H4	120.3
O1^i^—Gd1—O5	136.97 (13)	C5—C4—H4	120.3
O1—Gd1—O5^i^	136.97 (13)	N1—C5—C4	121.2 (5)
O1^i^—Gd1—O5^i^	137.36 (14)	N1—C5—C6	114.4 (4)
O5—Gd1—O5^i^	50.8 (2)	C4—C5—C6	124.4 (6)
O1—Gd1—O3	69.56 (13)	O1—C6—C7	122.4 (5)
O1^i^—Gd1—O3	116.68 (13)	O1—C6—C5	116.4 (5)
O5—Gd1—O3	68.60 (14)	C7—C6—C5	121.3 (5)
O5^i^—Gd1—O3	104.71 (14)	C8—C7—C6	120.2 (5)
O1—Gd1—O3^i^	116.68 (13)	C8—C7—H7	119.9
O1^i^—Gd1—O3^i^	69.56 (13)	C6—C7—H7	119.9
O5—Gd1—O3^i^	104.71 (14)	N2—C8—C7	128.4 (6)
O5^i^—Gd1—O3^i^	68.60 (14)	N2—C8—H8	115.8
O3—Gd1—O3^i^	172.95 (18)	C7—C8—H8	115.8
O1—Gd1—O2	74.31 (13)	N2—C9—H9A	109.5
O1^i^—Gd1—O2	145.72 (14)	N2—C9—H9B	109.5
O5—Gd1—O2	72.87 (13)	H9A—C9—H9B	109.5
O5^i^—Gd1—O2	71.10 (14)	N2—C9—H9C	109.5
O3—Gd1—O2	49.88 (13)	H9A—C9—H9C	109.5
O3^i^—Gd1—O2	127.03 (13)	H9B—C9—H9C	109.5
O1—Gd1—O2^i^	145.72 (14)	N2—C10—H10A	109.5
O1^i^—Gd1—O2^i^	74.31 (13)	N2—C10—H10B	109.5
O5—Gd1—O2^i^	71.10 (14)	H10A—C10—H10B	109.5
O5^i^—Gd1—O2^i^	72.87 (13)	N2—C10—H10C	109.5
O3—Gd1—O2^i^	127.03 (13)	H10A—C10—H10C	109.5
O3^i^—Gd1—O2^i^	49.88 (13)	H10B—C10—H10C	109.5
O2—Gd1—O2^i^	139.96 (19)	C1—N1—C5	118.3 (5)
O1—Gd1—N1	64.37 (14)	C1—N1—Gd1	124.5 (4)
O1^i^—Gd1—N1	90.57 (13)	C5—N1—Gd1	117.2 (3)
O5—Gd1—N1	128.14 (14)	C8—N2—C9	120.9 (6)
O5^i^—Gd1—N1	81.52 (14)	C8—N2—C10	121.7 (5)
O3—Gd1—N1	113.76 (14)	C9—N2—C10	117.4 (5)
O3^i^—Gd1—N1	68.23 (14)	O4—N3—O2	122.5 (6)
O2—Gd1—N1	73.16 (14)	O4—N3—O3	120.8 (6)
O2^i^—Gd1—N1	117.89 (15)	O2—N3—O3	116.7 (5)
O1—Gd1—N1^i^	90.57 (13)	O6—N4—O5^i^	123.4 (4)
O1^i^—Gd1—N1^i^	64.37 (14)	O6—N4—O5	123.4 (4)
O5—Gd1—N1^i^	81.52 (14)	O5^i^—N4—O5	113.2 (8)
O5^i^—Gd1—N1^i^	128.14 (14)	C6—O1—Gd1	125.4 (4)
O3—Gd1—N1^i^	68.23 (14)	N3—O2—Gd1	96.7 (3)
O3^i^—Gd1—N1^i^	113.76 (14)	N3—O3—Gd1	96.6 (3)
O2—Gd1—N1^i^	117.89 (15)	N4—O5—Gd1	98.0 (4)
O2^i^—Gd1—N1^i^	73.16 (14)	C11—O7—H7A	109.5
N1—Gd1—N1^i^	149.8 (2)	O7—C11—C12	132.5 (19)
O1—Gd1—N3^i^	134.19 (15)	O7—C11—H11A	104.1
O1^i^—Gd1—N3^i^	69.29 (14)	C12—C11—H11A	104.1
O5—Gd1—N3^i^	88.23 (15)	O7—C11—H11B	104.1
O5^i^—Gd1—N3^i^	69.43 (14)	C12—C11—H11B	104.1
O3—Gd1—N3^i^	151.60 (13)	H11A—C11—H11B	105.5
O3^i^—Gd1—N3^i^	25.09 (12)	C11B—O7B—H7B	109.5
O2—Gd1—N3^i^	139.72 (13)	O7B—C11B—C12B	122 (3)
O2^i^—Gd1—N3^i^	24.81 (12)	O7B—C11B—H11C	106.8
N1—Gd1—N3^i^	93.27 (16)	C12B—C11B—H11C	106.8
N1^i^—Gd1—N3^i^	93.04 (15)	O7B—C11B—H11D	106.8
N1—C1—C2	123.7 (6)	C12B—C11B—H11D	106.8
N1—C1—H1	118.2	H11C—C11B—H11D	106.7
C2—C1—H1	118.2	C11B—C12B—H12D	109.5
C3—C2—C1	118.2 (7)	C11B—C12B—H12E	109.5
C3—C2—H2	120.9	H12D—C12B—H12E	109.5
C1—C2—H2	120.9	C11B—C12B—H12F	109.5
C2—C3—C4	119.2 (6)	H12D—C12B—H12F	109.5
C2—C3—H3	120.4	H12E—C12B—H12F	109.5
C4—C3—H3	120.4		

Symmetry codes: (i) −*x*+1, *y*, −*z*+3/2.

### Hydrogen-bond geometry (Å, °)

**Table:**

*D*—H···*A*	*D*—H	H···*A*	*D*···*A*	*D*—H···*A*
O7—H7A···O3	0.82	2.26	3.084 (12)	179.
O7B—H7B···O3	0.82	2.19	3.00 (2)	168.
